# Hypokalemic Paralysis Complicated by Concurrent Hyperthyroidism and Chronic Alcoholism: A Case Report

**DOI:** 10.1097/MD.0000000000001689

**Published:** 2015-10-02

**Authors:** Ming-Hsien Tsai, Shih-Hua Lin, Jyh-Gang Leu, Yu-Wei Fang

**Affiliations:** From the Division of Nephrology, Department of Internal Medicine, Shin-Kong Wu Ho-Su Memorial Hospital (MHT, JGL, YWF); and Division of Nephrology, Department of Internal Medicine, Tri-Service General Hospital, National Defense Medical Center, Taipei, Taiwan (SHL).

## Abstract

Thyrotoxic periodic paralysis (TPP) is characterized by the presence of muscle paralysis, hypokalemia, and hyperthyroidism. We report the case of a young man with paralysis of the lower extremities, severe hypokalemia, and concurrent hyperthyroidism. TPP was suspected; therefore, treatment consisting of judicious potassium (K^+^) repletion and β-blocker administration was initiated. However, urinary K^+^ excretion rate, as well as refractoriness to treatment, was inconsistent with TPP. Chronic alcoholism was considered as an alternative cause of hypokalemia, and serum K^+^ was restored through vigorous K^+^ repletion and the addition of K^+^-sparing diuretics.

The presence of thyrotoxicosis and hypokalemia does not always indicate a diagnosis of TPP. Exclusion of TPP can be accomplished by immediate evaluation of urinary K^+^ excretion, acid-base status, and the amount of potassium chloride required to correct hypokalemia at presentation.

## INTRODUCTION

Hypokalemic paralysis, commonly observed in patients presenting to the emergency department, may be caused by neurologic, metabolic, or renal disorders. There is a high incidence of thyrotoxic periodic paralysis (TPP) among Asians, although the incidence has been recently increasing in Western countries because of global immigration.^1^ Excessive thyroid hormone levels can provoke abrupt intracellular shift of potassium (K^+^), thus causing paralysis. In cases of patients presenting with hypokalemic paralysis and hyperthyroidism, TPP is generally considered as the initial diagnosis. However, this diagnosis may be erroneous in rare cases owing to conditions that cause renal or nonrenal K^+^ loss. Incorrect diagnosis may cause the implementation of an inappropriate strategy of K^+^ repletion and recurrent hypokalemia. The use of simple, fast, and inexpensive tests of blood and urine electrolytes, and acid-base status may aid in differentiating such disorders.^2,3^ This could be illustrated by the following young male presenting with hypokalemic paralysis, who was initially misdiagnosed as TPP, but established to have the etiology of chronic alcoholism.

## CASE REPORT

A 33-year-old Chinese man with a family history of hyperthyroidism presented to the emergency department of our institution with a 5-day history of progressive muscular weakness and paralysis of the lower extremities. Symptoms such as nausea, vomiting, diarrhea, or lower-extremity numbness were absent. There was no history of recent strenuous exercise or diuretic use. The patient was a chronic alcoholic for more than 10 years and reported excessive drinking the week before symptom onset. On physical examination, blood pressure was 116/64 mm Hg, heart rate was 82 beats/min, respiratory rate was 22 breaths/min, body temperature was 36.8 °C, and the patient had symmetrical flaccid paralysis of the lower extremities with areflexia. The remainder of the physical examination was unremarkable.

**FIGURE 1:**
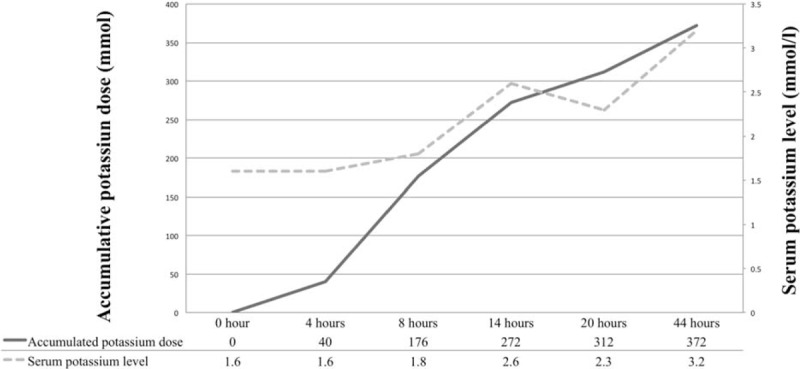
Relationship between serum potassium level and dose of potassium supplementation. The patient's serum level of potassium returned to normal in 44 h after supplementation with 372 mmol of potassium chloride.

The biochemical studies are shown in Table 1. Severe hypokalemia (1.6 mmol/L) with metabolic alkalosis (bicarbonate 36 mmol/L) was the prominent finding. Serial urine electrolytes examined during admission indicated an inconsistent K^+^ excretion rate (transtubular potassium gradient [TTKG] 7.06 and urinary K^+^-creatinine ratio [K^+^/Cr] 3.97 on day 1, but TTKG 0.84 and urinary K^+^/Cr 1.84 in day 2). Hormone profile, including thyroid-stimulating hormone (<0.005 mIU/L; reference range 0.27–4.2), free thyroxine (fT_4_) (24 pmol/L; reference range 11.6–21.2), and cortisol (507.6 nmol/L; reference range 171.0–535.2), indicated hyperthyroidism. The initial diagnosis was TPP, and nonselective β-blocker therapy (propranolol 60 mg/day) with cautious K^+^ supplementation was administered. However, serum K^+^ level remained unchanged for several hours (Figure 1). Because the patient also had metabolic alkalosis and high urinary K^+^ excretion, which suggested a severe K^+^ deficit associated with chronic alcoholism rather than acute hypokalemia with relatively normal acid-base status as seen in TPP, he received a larger dose of K^+^ supplement along with the addition of spironolactone. A total of 372 mmol of potassium chloride (KCl) was needed to achieve normal serum K^+^ concentration and restore his muscle strength (Figure 1). Three days later, the patient had normal serum K^+^ level and did not develop hyperkalemia. He was discharged with antithyroid treatment and was persuaded to cease alcohol consumption. At the 1-month follow-up, his serum K^+^ concentration was 3.6 mmol/L without KCl supplementation.

**TABLE 1:**
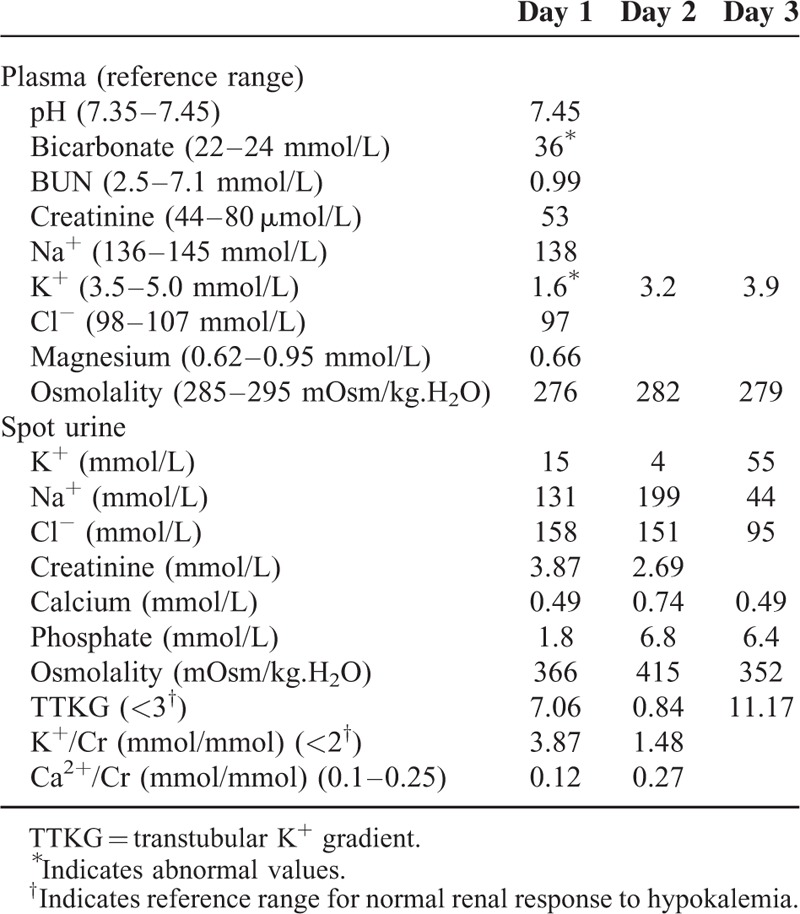
Serum and Urinary Laboratory Data During Admission

## DISCUSSION

Hypokalemia is a common but potentially life-threatening electrolyte abnormality because it may induce cardiac arrhythmia and respiratory failure. The most essential role of K^+^ is to keep the transmembrane's electrochemical gradient. Short-term homeostasis of the serum concentration of K^+^ is determined on a minute-to-minute basis by the K^+^ shifting between intracellular and extracellular fluid compartments. Nevertheless, long-term homeostasis of serum K^+^ level depends on a day-to-day basis by the regulation of renal K^+^ excretion. Therefore, the etiologies of hypokalemia usually are a shift of K^+^ into cells and/or excessive renal or extrarenal K^+^ loss.^4,5^ Hypokalemic periodic paralysis contains any hypokalemic disease caused by acute shift of K^+^ into cells. Two most common subgroups are identified. One is TPP, which is mainly observed in Asians, and the other is familial periodic paralysisis, which is induced by calcium or sodium channelopathies of skeletal muscle and predominantly seen in Caucasians.^1,6^ TPP can manifest with hyperthyroidism of any etiology. Hyperthyroidism can induce a hyperadrenergic state.^7^ Stimulation of β2-adrenergic receptor in muscle cells may directly promote cellular K^+^ uptake by increasing intracellular cyclic adenosine monophosphate, which thus can lead to activation of the Na^+^-K^+^-ATPase pump.^8^ Moreover, thyroid hormone has a similar chemical structure to that of catecholamines, which can directly activate the Na^+^-K^+^-ATPase pump and concurrently increase the number and sensitivity of β-receptors.^9^

Chronic alcoholism is usually associated with electrolyte abnormalities and metabolic alkalosis. Although disturbances in magnesium and phosphorus homeostasis can occur in these patients, hypokalemia is found more frequently.^10,11^ The causes of hypokalemia in alcoholism include poor intake, gastrointestinal loss (diarrhea or vomiting), hypomagnesemia, and renal tubular dysfunction. The exact mechanism underlying renal K^+^ loss is unclear, but hypomagnesaemia, which may open K^+^ channels in the luminal membrane of the distal tubules and increase K^+^ excretion, may be involved.^12^ Accordingly, in the hypokalemic state associated with chronic alcoholism, the urinary K^+^ excretion rate is inconsistent, and may be high because of renal tubule dysfunction or low because of gastrointestinal K^+^ loss or poor K^+^ intake. In the present case, the serum magnesium level was normal and serum K^+^ was elevated merely by K^+^ supplementation without additional magnesium administration, suggesting that magnesium played a minor role in this condition.

**FIGURE 2:**
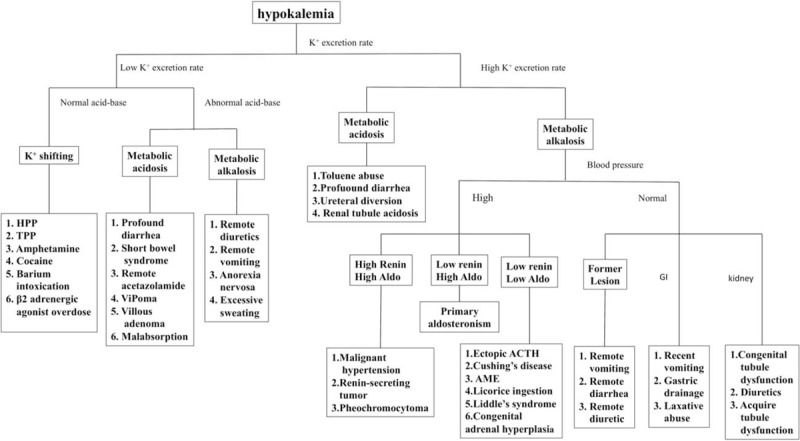
Approach to hypokalemic patients.^2–4,13,14^ Abbreviation: Aldo, aldosterone; ACTH, adrenocorticotropic hormone; AME, apparent mineralcocorticoid excess syndrome.

The causes and differential diagnosis of hypokalemia are shown in Figure 2. Assessment of urinary K^+^ excretion rate and blood acid-base state is paramount to help determine the cause of hypokalemic paralysis. TTKG (reference range 3) or spot urinary K^+^-creatinine ratio (reference range 2.0 mmol/mmol) can be used as an index of urinary K^+^ excretion rate. If a low urinary K^+^ excretion rate is noted, extrarenal loss or hypokalemic periodic paralysis should be considered. In HPP, including TPP, the acid-base state is relatively normal, whereas in the presence of extrarenal causes such as gastrointestinal disorder, metabolic alkalosis or acidosis usually occurs.^13,14^ If a high urinary K^+^ secretion rate is present, abnormal values of blood acid-base state and blood pressure provide useful information. Coexisting metabolic acidosis suggests the presence of renal tubular acidosis (RTA), toluene use, severe diarrhea, or ureteral diversion. The presence of hypertension with metabolic alkalosis indicates excess of mineralocorticoids. The presence of normal blood pressure and metabolic alkalosis indicates genetic renal tubulopathy (Gitelman syndrome and Bartter syndrome), severe diarrhea, vomiting, diuretic use, or alcoholism-induced renal tubular dysfunction.^2–4^

In the present case, the first spot urine analysis performed at the emergency department showed a high urinary K^+^ excretion rate. Vomiting, diarrhea, and diuretic use were absent. A serial urinary electrolyte excretion did not support the diagnosis of genetic renal tubular disorders such as Bartter and Gitelman syndrome, with persistent high urinary NaCl excretion and divalent abnormality in blood and urine. TPP was highly suspected solely because of the presence of hyperthyroidism and hypokalemia. However, β-blocker administration to treat the acute onset of TPP coupled with K^+^ supplementation for several hours failed to elevate serum K^+^ concentration, inconsistent with the typical course of TPP. The recognition of concurrent chronic alcoholism and the presence of metabolic alkalosis, high renal K^+^ secretion rate, and low urine calcium/phosphate ratio (usually more than 1.7 in TPP)^2^ in the first spot urine assessment prompted us to associate the severe K^+^ deficit with chronic alcoholism as the primary cause. TPP can be triggered by alcohol consumption.^15^ However, in the present case, increase in the dose of K^+^ supplement did not result in development of rebound hyperkalemia on recovery, as is usually seen in most cases of TPP^16^; this was an additional indicator that the diagnosis of TPP was incorrect.

In conclusion, a provisional diagnosis of TPP was premature when facing a patient presenting with hypokalemia and hyperthyroidism. A detailed history, careful physical examination, urinary K^+^ excretion rate, and blood acid-base state may help determine the cause of hypokalemia.
